# Ethiopian primary healthcare clinical guidelines 5 years on—processes and lessons learnt from scaling up a primary healthcare initiative

**DOI:** 10.1136/bmjgh-2023-013817

**Published:** 2024-10-28

**Authors:** Aklog Getnet Kibret, Wondosen Mengiste Belete, Charlotte Hanlon, Israel Ataro, Kiflemariam Tsegaye, Zelalem Tadesse, Meseret Feleke, Megersa Abdella, Meseret Wale, Kassahun Befekadu, Alemayehu Bekele, Daniella Georgeu-Pepper, Christy-Joy Ras, Lara R Fairall, Ruth Vania Cornick

**Affiliations:** 1 Community Engagement and Primary Health Care Lead Executive Office, Ethiopia Ministry of Health, Addis Ababa, Ethiopia; 2 Institute of Psychiatry, Psychology and Neuroscience, Health Service and Population Research Department, Centre for Global Mental Health, King's College London, London, UK; 3 Centre for Innovative Drug Development and Therapeutic Trials for Africa (CDT-Africa), College of Health Sciences, Addis Ababa University, Addis Ababa, Ethiopia; 4 Knowledge Translation Unit, Department of Medicine, University of Cape Town, Cape Town, South Africa; 5 School of Life Course & Population Sciences, King's College London, London, UK

**Keywords:** Health systems, Health systems evaluation, Global Health, Public Health

## Abstract

Many effective health system innovations fail to reach those who need them most, falling short of the goal of universal health coverage. In the 5 years since the Federal Ministry of Health in Ethiopia localised the Practical Approach to Care Kit (PACK) programme to support primary care reforms, PACK has been scaled-up to over 90% of the country’s primary care health centres. Known as the Ethiopian Primary Healthcare Clinical Guideline (EPHCG), the programme comprises a comprehensive, policy-aligned clinical decision support tool (EPHCG guide) and an implementation strategy to embed comprehensive, integrated care into every primary care consultation for individuals over 5 years of age, while addressing barriers to streamlined primary healthcare delivery. We describe the components of the EPHCG programme and the work done to establish it in Ethiopia. Yamey’s framework for successful scale-up is used to examine the programme and health system factors that enabled its scale-up within a 5-year period. These included high-level ministry leadership and support, a cascade model of implementation embedded in all levels of the health system, regular EPHCG guide and training material updates and strategies to generate stakeholder buy-in from managers, health workers, patients and communities. Challenges, including stakeholder resistance, training fidelity and quality and procurement of medicines and diagnostic tests, are described, along with efforts to resolve them. Insights and learnings will be of interest to those implementing PACK programmes elsewhere, and managers and researchers responsible for design and delivery of health systems strengthening innovations at scale in low-income and middle-income countries.

**SUMMARY BOX**

- There is a global imperative to reorient health systems towards primary healthcare; however, many effective healthcare innovations fail to scale-up to reach those that need them most.
- The Practical Approach to Care Kit (PACK) is a strategy to support the delivery of primary healthcare in resource-limited settings.
- In Ethiopia, an adaptation of PACK, the Ethiopian Primary Healthcare Clinical Guideline (EPHCG) programme has been scaled-up to 92% of primary care health centres over a 5-year period.
- Key programme and system elements that supported this scale-up included strong leadership, extensive stakeholder engagement, regular update of clinical content, cascade implementation and incorporation of programme standards into routine monitoring and evaluation activities.
- The paper will contribute to more robust evaluation of the EPHCG programme scale-up and has practical relevance for those embarking on PACK implementations or considering strategies to support primary care initiatives.

## Introduction

There is a global imperative to reorient health systems towards primary healthcare (PHC).^1 2^ Governments, donors, health systems and communities can ill-afford investments in innovations that work but never go to scale.^3^ The science of scale-up aims to determine and evaluate elements of health system implementation that ensure an innovation is widespread and sustained.^4^

Ethiopia’s Federal Ministry of Health (FMoH) is reforming its health services to achieve United Nations Sustainable Development Goal (SDG) 3.8 of universal health coverage by 2030.^5^ The 20-year Health Sector Development Programme impacted health and nutrition outcomes—improving life expectancy, under-five mortality, maternal health, and prevention and control of major communicable diseases. However, the impact of non-communicable diseases (NCDs) and mental illness remained high, reflecting shifting disease burdens globally.^6^ The next phase of Ethiopia’s health sector strategy is committed to tackling this double burden.^7^

The Health Sector Transformation Plan 1 (2015/16–2019/20)^8^ guided Ethiopia’s health sector reforms, aligned with SDG 3.8.^9^ The Practical Approach to Care Kit (PACK) was one of the initiatives adopted by the FMoH in 2016 to support this vision in PHC^10^ and link policy to practice.^11^

The PACK programme is a health system strengthening strategy implemented in low-income and middle-income countries (LMICs), including South Africa and Brazil^12^ that aims to streamline PHC delivery to be policy-aligned, integrated and coordinated. Evaluations show consistent and sustained improvements on quality-of-care and health outcomes.^13^,^15^ The PACK guide is concise (160 pages) yet comprehensive, supporting clinical decision-making by frontline health workers.^16^ Its 3500 recommendations draw on WHO guidance. Cased-based training encourages guide use within clinical consultations, focusing on priority clinical issues and structural barriers to implementation.^17^ Educational outreach training sessions use a cascade implementation model, with online and hybrid delivery since the COVID-19 pandemic.^18 19^

Ethiopia’s healthcare governance structure includes administrative and fiscal decentralisation to regional health bureaus, zonal health departments and district (woreda) health offices. A woreda-based PHC system uses a network of community health posts, health centres and primary hospitals. Health centres provide preventive, curative and usually only minor surgical services. They serve as referral centres and practical training sites for health extension workers staffing health posts.

The FMoH worked with PACK’s developers, the Knowledge Translation Unit (KTU) of the University of Cape Town, to localise the programme to local clinical policy, medication lists, disease burden, resource constraints and health worker scope of practice. This process is described elsewhere.^20^ Launched as the Ethiopian Primary Healthcare Clinical Guidelines (EPHCG), it has rolled out to over 90% of health centres and forms a core component of PHC system governance.

This practice paper is one in a collection describing PACK’s role as part of health system reforms in LMICs. It describes the work to establish the EPHCG programme into PHC services and scale it up, including challenges encountered and responses to these. We identify programme and contextual elements that led to its successful scale-up, sharing insights relevant to those working in Ethiopian PHC, embarking on PACK implementations elsewhere or considering strategies to scale-up other initiatives to achieve universal health coverage.

### Components of the EPHCG programme

The components of the EPHCG programme comprise those localised from the PACK programme along with those developed locally in response to need and challenges outlined below. They include the guide (printed and app versions), patient information booklets, training curriculum, programme and materials, implementation frameworks and strategies, and monitoring and evaluation tools. Table 1 provides a list of the 15 tools, with further detail included in online supplemental file 1.

**Table 1:** Components of the EPHCG programme

Item	Purpose
Clinical tools	
EPHCG guide—hard copy version	To provide consolidated and user-friendly national policy-aligned clinical decision support for health professionals working in health centres.
EPHCG guide—app version—Android and desktop applications	Developed to circumvent printing costs and make the EPHCG guide more widely available.
EPHCG-linked patient information booklets	To strengthen self-management for people with NCDs and mental health conditions treated through the EPHCG.

EPHCG training materials and programme	
Training curriculum	To familiarise users with EPHCG guide structure and features while focusing on clinical priorities and potential systems bottlenecks to embed its use in clinical practice.
Master Trainer Manual	To support master trainers to deliver a facility trainers’ training workshop and coordinate programme implementation at regional, zonal and woreda level.
Facility Trainer Manual	To guide facility trainers to deliver onsite training sessions to health professionals.
Training resources	To support interactive sessions, allowing trainees to easily grasp EPHCG concepts in a fun, practical and visually compelling way.
Online training platform	To enable provision of EPHCG training to remote health workers, reduce training costs, streamline EPHCG guide update trainings, standardise the training curriculum and enable training of new recruits when it is impractical to organise onsite sessions for fewer than three participants.
EPHCG online facilitators’ manual	To guide EPHCG online facilitators to facilitate online training sessions and monitor training progress.
Primary healthcare clinical communication skills training manual and training programme	To support health professionals to deliver person-centred care while using the EPHCG guide.
EPHCG-linked quality improvement training programme	To equip health professionals in health centres with skills and tools to perform quality improvement initiatives while implementing EPHCG, focusing on NCDs and mental health conditions.

Implementation tools and strategies	
EPHCG Implementation Manual	To standardise EPHCG programme implementation across all health centres. The manual also addresses a monitoring framework for programme managers at all levels.
EPHCG implementation support strategies	To support the EPHCG training cascade and to sustain woreda and facility-level motivation to continue with EPHCG programme implementation.

Monitoring and evaluation	
EPHCG training record	To monitor adherence of health centres to training model at facility level.
EPHCG informing Clinical Audit Quality Indicators	To provide a standard of care for clinical auditing, against which health centre clinical practices are measured. To standardise EPHCG implementation across all health centres and to improve health worker adherence to the EPHCG guide.

EPHCG, Ethiopian Primary Healthcare Clinical Guideline; NCD, non-communicable disease.

#### EPHCG programme implementation

**Figure 1:**
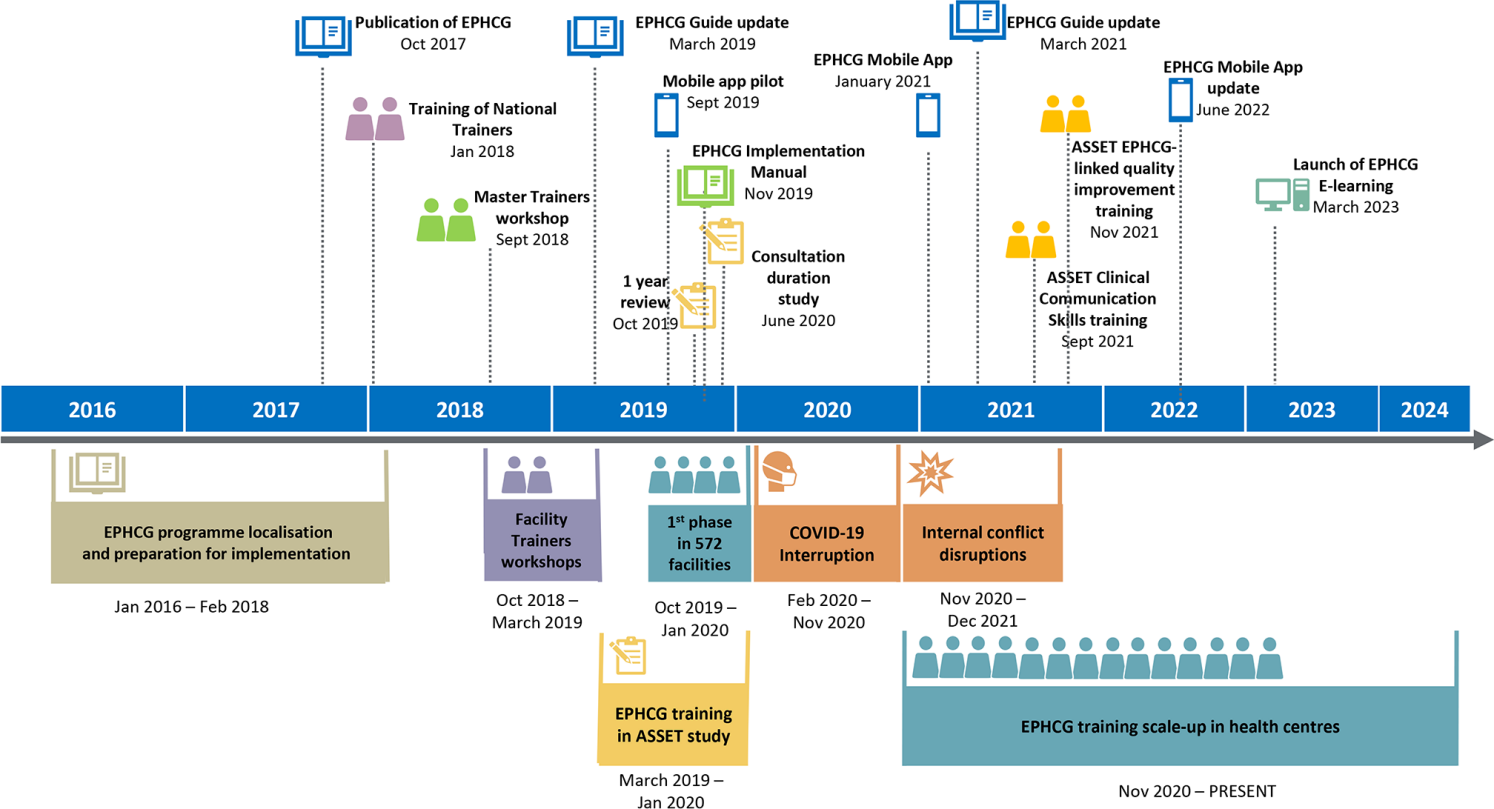
Timeline of EPHCG implementation activities. EPHCG, Ethiopian Primary Healthcare Clinical Guideline.

Figure 1 gives a visual timeline from 2016 to 2023 of EPHCG implementation activities, and key programme and external events that influenced scale-up.

EPHCG was initially implemented in 572 better performing and well-resourced health centres across the country. A mixed methods evaluation in 18 health centres identified barriers and enablers to EPHCG implementation with a focus on NCDs and mental health^21^ and informed training around clinical communication skills and quality improvement to support EPHCG implementation. After incorporating feedback from this phase,^22^ national scale-up proceeded, interrupted only during the COVID-19 pandemic and by internal conflict in certain regions.^23^ 92% of all health centres (n=3574) received EPHCG training. In the absence of country-wide aggregated training data, this reach is estimated as 10 health professionals per health centre; approximately 35 740 in total.

**Figure 2:**
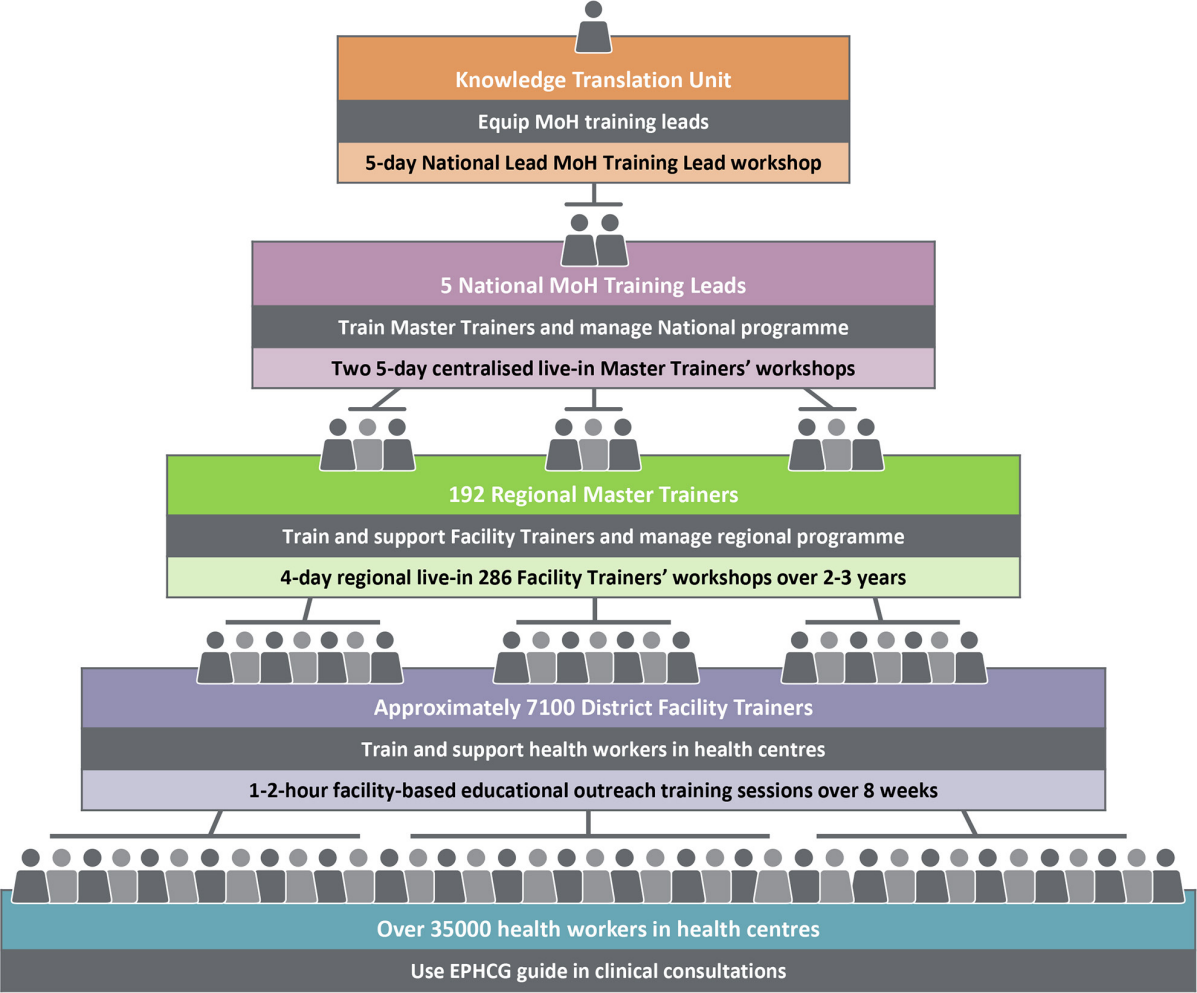
Scaling up the EPHCG programme using a cascade training model. EPHCG, Ethiopian Primary Healthcare Clinical Guideline.

Implementation used a cascade model for scale-up, depicted in figure 2. National-level training was provided by five FMoH training leads, supported by the KTU, to 192 representatives of all regional health bureaus, FMoH directorate staff and partner organisations. These master trainers provided training workshops to approximately 7100 facility-based trainers in their respective regions. Clinicians and managerial staff working in woreda health offices and hospital staff also attended to ensure mentorship, monitoring and evaluation. Generally, two facility trainers delivered on-site training to all health officers, nurses, midwives and where present, non-specialist doctors, in their health centre over an 8-week period. Pharmacists, laboratory technologists and supporting staff received EPHCG programme orientation.

Strategies were initiated to support the training cascade and sustain woreda and facility motivation to continue implementation:

- Supportive supervision and mentorship from facility colleagues, woreda health offices and catchment hospitals
- Using EPHCG programme indicators to recognise efforts of best-performing health centres
- Experience-sharing visits to best-performing health centres
- A messaging platform to follow regional training rollout, share health centre experiences, provide weekly updates of resources, guidance and a forum for questions.
- Community awareness about EPHCG through existing platforms like town hall meetings, facility health education sessions, multimedia and radio.
- Two-yearly EPHCG guide updates to reflect evolving evidence, feedback from end-users, and updated or new national guidelines

Box 1 provides a vignette of EPHCG implementation in one health centre.

Box 1 **Vignette: EPHCG implementation in Gereno Health Centre, Gurage zone, Ethiopia**

(Adapted from Ethiopian Federal Ministry of Health Special Bulletin 21^st^ Annual Review Meeting 2019)^43^

The Gereno Health Centre was one of 16 in the Gurage zone that participated in the first phase of EPHCG implementation in 2019 and was included in the ASSET study. It serves a rural population of around 21 000 and many people co-ordinate their visits with a weekly market day in the town. It also provides accommodation for expectant mothers to support attended deliveries as road infrastructure is too limited to guarantee rapid transport during an emergency.

The health centre completed the EPHCG onsite training sessions within 11 weeks, with all 10 health professionals participating. They started using the EPHCG guide during every consultation after the first session in all relevant outpatient department services. From the outset, considerable effort was made at woreda level, within the health centre and at community level to support EPHCG implementation, with Woreda health officers advocating EPHCG guide use in various forums. The health centre delivered awareness raising sessions in public forums, and community-based health extension workers familiarised households with the benefits of being treated by health workers using the guide. This helped mitigate the anxiety health professionals initially felt when opening the EPHCG guide in front of patients.

The format of short, weekly group sessions alternating with practice inspired the staff to establish a weekly clinical forum after their initial training was completed. During these sessions, they shared experiences and knowledge, presented challenging clinical cases and worked through how they would manage such cases using the EPHCG guide. The sessions also provided space to troubleshoot day-to-day challenges around laboratory tests, medications and advice-giving as a team. The health centre head noted, ‘The continued clinical discussion forum significantly reduced negligence among our staff. Our health professionals are careful because everybody is ready to be reviewed in the discussion. Every professional is scrutinised. It helps us to improve our knowledge and skills. Previously, there is not any such forums in the health centre.’ This was echoed by a health worker who also highlighted the value of a safe space to share difficulties: ‘The weekly clinical forum helps us to improve collaboration among health workers, own our problems and solve it. In addition, it boosts our confidence to understand the (EPHCG) guideline.’

The group nature of the training proved central to expanding capacity and standardising care within the health centre, an often-overlooked component of large scale-up initiatives. The health centre head added: ‘Before EPHCG implementation, patients prefer two or three professionals, and they asked these professionals by name. If they did not get one of them, they would return and came back some other day. Now that trend is changed because EPHCG standardised the care given in the health centre, and patients are treated by any available health workers*.*’

A challenge encountered during EPHCG implementation in the health centre was that patients could not receive the prescribed drugs in the health centre as per the EPHCG guide because the previous list of drugs recommended by the health centre’s Drug and Therapeutic Committee (DTC) was very limited, containing just 79 items. To solve this challenge, the DTC revised the drug lists based on the medications contained in the EPHCG guide and top 10 diseases of the area. Accordingly, the list increased to 174 drugs. The list of laboratory tests was also expanded to align with the EPHCG guide. The health centre worked to make all drugs listed available in its pharmacy, purchasing drugs from the MoH Ethiopian Pharmaceutical Supply Agency (EPSA), and where items were not available, from private pharmaceutical vendors. Drug procurement and prescribing practices were influenced by EPHCG implementation, including reductions in polypharmacy per patient, improvements in essential drug availability and decreases in drug wastage, with full utilisation of appropriately procured drugs reported during the first 6 months of EPHCG implementation.

Key factors in the successful and sustained EPHCG implementation in Gereno included support provided by the Woreda Health Office advocating for the programme among health workers and the public, the establishment of a regular clinical discussion forum and improvements to drug procurement processes.

### Enablers and challenges of EPHCG programme scale-up

There are many frameworks and models for conducting and evaluating health intervention scale-up.^24^ We chose Yamey’s framework to explore factors that contributed to EPHCG programme’s scale-up, as it was developed for LMIC settings and instead of having a disease-specific focus, applies to general health interventions.^25^ Its elements are outlined in table 2 and its application to EPCG scale-up along with challenges and learnings, discussed below.

**Table 2:** Yamey framework for global health intervention scale-up^25^

Category	Factors supporting scale-up
Attributes of innovation	Simplicity
	Scientifically robust technical policies
Attributes of implementers	Strong leadership and governance
	Engaging local implementers and other stakeholders
	Using both state and non-state actors as implementers
Chosen delivery strategy	Applying diffusion and social network theories
	Cascade and phased approaches to scale up
	Tailoring scale-up to the local situation and decentralising delivery
	Adopting an integrated approach to scale-up
Attributes of adopting community	An engaged activated community
Sociopolitical context	Political will and national policies
	Country ownership
Research context	Incorporating research into implementation (learning and doing)

#### Attributes of the innovation

Simplicity. While implementing a new, multicomponent programme into a complex health system is not a simple process, there are elements of the EPHCG programme that embrace simplicity to encourage its adoption into PHC practice. The curriculum scaffolds from simple to complex clinical scenarios and is limited in scope, focusing on service and clinical priorities.

The EPHCG guide consolidates a plethora of clinical guidelines and protocols to include only essential detail, using checklists and algorithms for ease-of-use and avoiding medical jargon. At times, the FMoH team experienced technical difficulties adjusting the guide’s content during updates as it interfered with its structure and page numbering. This issue has not yet been resolved but could be addressed in the future by KTU mentorship to develop in-house clinical editorial capacity to manage guide updates.

Cost and quality issues with printed copies of the guide prompted an app version, which may facilitate user-uptake and more regular publication of updates.

Scientifically robust technical policies. The EPHCG guide aligns to local clinical policy and is drawn from an evidence-based PACK guide template. Two-yearly EPHCG guide updates ensure ongoing alignment with evidence and latest policy.

### Attributes of the implementers

An accepted principle of scale-up strategies is the requirement for broad stakeholder engagement.^26^

Strong leadership and governance. The EPHCG programme received ongoing and visible support from FMoH leadership from inception. This helped sustain prioritisation, maintain close follow-up and ensure equity of scale-up across all woredas.

Engaging local implementers and other stakeholders. The cascade training model employs health system actors and implementers at all levels—federal, regional health bureau, woreda and within facilities.

Using both state and non-state actors as implementers. The FMoH worked closely with academic, donor and non-profit organisations that provided financial, evaluation and technical assistance to support EPHCG development and implementation activities, including training delivery, printing of guide and training materials, and development of EPHCG app and training manuals.

### Chosen delivery strategy

Applying diffusion and social network theories. While EPHCG programme scale-up did not explicitly conform to specific diffusion or social network theories, the process inherently drew on them^27^:

- The EPHCG programme addressed the needs of adopters and was compatible with both health system and staff.
- Experience and achievements of early adopters were celebrated and shared.
- Communication about EPHCG implementation used existing health system and community channels.
- Implementers were existing members of the health system and thus trusted as programme representatives.

Cascade and phased approaches to scale-up. The MoH conducted a review following the first phase of implementation in 572 facilities, with lessons learnt integrated in subsequent phases.^23^

The cascade model of training enabled EPHCG scale-up to over 90% of health centres over 5 years and establishment of a support platform for ongoing implementation. This model also facilitated PACK scale-up in South Africa as well as other initiatives elsewhere.^28^ The scale-up mechanism suited the health system’s tiered structure and appeared to enhance relationships between FMoH and regional health bureaus and create a sense of ownership and accountability at all levels.

The cascade model was frustrated by varied facility trainer capacity and motivation to deliver EPHCG training, and the turnover of trainers. The FMoH trained additional trainers to replace those no longer active and used the cascade to provide supportive supervision for trainers.

Additional budget was required to roll-out a nationwide programme. While the cascade model of training was not more expensive than delivering other training programmes (staff involved were all government employees), the cost was still considerable as it aimed to be nationwide over a short period and occur alongside existing programme-focused trainings. To address this, some diversion of budget occurred from programme case teams to the training and supervision of EPHCG.

These challenges prompted development of an online EPHCG curriculum on the FMoH e-learning platform as a standardised training that did not require facilitation from a trainer and which clinicians could access when it suited them. E-learning had a cost advantage for the FMoH over a face-to-face model, but uptake was limited initially as providers were unwilling to pay for their own internet use. Variable computer skills among users and poor internet access were other challenges. Providing internet access through existing in-facility health information system platforms, basic computer skills training and offering credit hours for re-licensing requirements helped to address these barriers.

Tailoring scale-up to the local situation and decentralising delivery. Decentralising delivery to woreda and facility level aligned with woreda-based healthcare planning. While disseminating clinical guidance and delivering training does not necessarily lead to use of that guidance in practice, educational outreach is a proven methodology for changing clinical practice.^29^ The EPHCG educational outreach training methodology and programme structure proved acceptable and feasible, despite the pedagogical shift from expert-delivered lectures to interactive, case-based training conducted by non-experts. Team-based sessions ensured training saturation within a facility, and by boosting the culture of regular clinical forums among health centre staff, reinforced the concept of teams delivering care rather than individuals. The Gereno health centre vignette (Box 1) illustrated this, describing patients satisfied to be seen by any team member following EPHCG introduction.

Shifting from ‘hotel-based’ training increased health centre capacity to train health professionals in EPHCG when needed, not just when external partners, the FMoH or regional health bureaus fund it. This is essential given high PHC professional turnover and need for continuous updates.

There was, however, some resistance from health centre clinicians to the unfamiliar onsite training model due to workload pressures and in some instances, lack of managerial support to participate. A significant factor was the absence of a ‘per diem’ financial incentive for attending on-site training (unlike for off-site training) which has been described elsewhere as having a negative effect on organisational culture.^30^

In response, experience-sharing programmes were introduced which included selection of best-performing health centres per region to recognise their efforts. Mentorship from experienced clinicians, assessing adherence to EPHCG programme standards using case management checklists and awarding trainees continuing education units for completing the training also provided incentive.

Adopting an integrated approach to scale-up. The EPHCG programme was integrated into existing PHC services, using existing staff and resources, and complementing or replacing vertical programmes. The educational outreach approach subsequently informed the implementation strategy for quality improvement and clinical communication skills programmes.^31^

However, the integrated, comprehensive approach of EPHCG challenged prevailing clinical governance at federal, regional health bureau and health facility level (including that of implementing partners and donors) that supports a programme approach focusing on priority health issues, for example, mental health or NCDs. A common concern was that a comprehensive, integrated approach would undermine the purpose and efforts of separate condition-focused programmes, a perspective described as a barrier to successful PHC delivery.^32^ Endorsement of the EPHCG programme as a ministry priority helped support the EPHCG approach, with budget diverted from programme case teams to EPHCG training and supervision.

Despite extensive efforts to tailor EPHCG guide recommendations to local policy, health professionals were at times unable to implement them due to resource allocation and supply issues. This experience is borne out in a systematic review of studies exploring barriers to clinical guideline use in LMICs as a key factor for non-adherence.^33^ Due to system level bottlenecks, most health centres do not have laboratory tests and drugs as per minimum health centre standards reflected in the EPHCG guide.^34^ This prompted some improvements in procurement system processes at facility and regional levels, guiding the supply chain system to avoid purchase of non-essential drugs or diagnostic equipment. However, challenges remain.

### Attributes of the ‘adopting’ community

An engaged activated community. In general, PHC clinicians and managers were willing to engage with the EPHCG programme as it speaks to their need to support delivery of comprehensive care rather than to donor-driven or disease-siloed interests. Clinician concerns that using the EPHCG guide takes too long compared with usual approach prompted a preliminary FMoH evaluation of this issue which suggested that the extra consultation time when using EPHCG was not considerable. This issue requires further evaluation.

Initially, many health workers avoided using the guide in front of patients for fear of appearing unprofessional, a concern we have repeatedly encountered around use of PACK guidance in clinical consultations. This is of interest, as while the literature exploring barriers to clinician adherence to guidance includes sensitivities around it threatening their professional autonomy, we have not found fear of appearing unprofessional reported elsewhere.^33^ Patient buy-in, generated by community-level awareness campaigns (with some patients even requesting care according to the EPHCG guide), appeared to increase health professional comfort to use it during consultations. This experience echoes that of PACK implementers in Nigeria. As an essential component of PHC,^35^ community engagement has in this instance supported EPHCG implementation; its potential to support other elements of the programme, such as clinical priority setting or communication, remains to be explored.

Experience-sharing initiatives created opportunities for recognition of performance and appeared to improve motivation and generate competitive spirit among health professionals, health centres and regions, particularly during early implementation.

### Socio-political context

Political will, national policies and country ownership. Success of a system level change depends on every level of leadership.^36^ The FMoH designated EPHCG a flagship initiative of ministry leadership, renaming PACK to become EPHCG. This helped to ensure ownership and buy-in at each level of the health system.

### Research context

#### Incorporating research into implementation (learning and doing)

The FMoH has established a learning health system that uses a phased approach to implementation and draws on routinely collected data about EPHCG performance. This supports delivery of quality care, identification of programme challenges and limitations and informs programme adjustments as required.^4^ The National Health Insurance Agency adopted the EPHCG guide as an audit tool to determine health centre eligibility for reimbursement for services. Incorporating standards of EPHCG as quality indicators in monitoring and evaluation frameworks appeared to improve adherence to the guide. A pilot project currently underway in selected health centres is the integration of EPHCG into existing electronic medical record systems which might facilitate these monitoring and evaluation activities. In addition, FMoH collaborates with academic partners to identify bottlenecks and develop, evaluate and optimise health system strengthening interventions to support EPHCG programme scale-up.^21^

## Conclusion

Ethiopia is well known for sustaining successful health intervention scale-ups; the 20-year-old FMoH health extension worker programme a key example.^37^ The EPHCG programme scale-up addressed key barriers to implementation of WHO’s Integrated Management of Childhood Illnesses (IMCI) in over 100 countries—where, as described by Chopra and colleagues, the IMCI guide was inadequately localised, staff training insufficient, supervision and referral mechanisms lacking and health services poorly used.^38^

Positioning the EPHCG as a core component of Ethiopia’s primary care reforms helped embed the programme into service delivery efforts^11^ and mirrors PACK implementation in South Africa where it forms part of the ‘Ideal Clinic’ initiative.^39 40^

The integrative, system strengthening approach of EPHCG had a good fit with a PHC-orientated health system and was welcomed by policymakers, planners and providers.^1^ Collaborative implementation research programmes, broad stakeholder participation and strong country ownership provided fertile ground for implementation strategies and innovations to strengthen EPHCG scale-up, creating the foundations of a learning health system.^41 42^ Ongoing work will be needed to evaluate how to optimise the programme, sustain its implementation beyond 5 years and understand how it supports efforts to orientate the system towards PHC.

EPHCG programme scale-up provides an illustrative case study of strategies and elements for reproducing an effective health systems innovation and taking it to scale. It may help those planning a scale-up of the PACK programme or other innovations to reorient their health system towards PHC.

## Supplementary material

10.1136/bmjgh-2023-013817 online supplemental file 1

## Data Availability

Data sharing not applicable as no datasets generated and/or analysed for this study.
