# Supplementary material for: Ethiopian primary healthcare clinical guidelines 5 years on—processes and lessons learnt from scaling up a primary healthcare initiative
Source: BMJ Glob Health. 2024 Oct 28;9(Suppl 3):e013817. doi: 10.1136/bmjgh-2023-013817 (PMC11664385; doi:10.1136/bmjgh-2023-013817)
Supplement: online supplemental file 1 [file bmjgh-9-Suppl_3-s001.pdf]

Supplemental file 1: Components of the EPHCG programme

Item	Purpose	Description	User/audience	Derived from PACK Global package or local innovation	Date developed or updated
**Clinical tools**					
**EPHCG guide – hard copy version**	To provide consolidated and user-friendly national policy-aligned clinical decision support for health professionals working in health centres.	• 162-page comprehensive, integrated clinical content with 99 adult symptoms, 37 symptoms in the child five years and older and 35 conditions requiring long-term care, including communicable (TB, HIV), non-communicable, mental health and women's health conditions.<br>• The hard copy version is ring-bound and printed on durable paper which supports daily use in clinical practice and makes it easy to navigate between pages.	Facility trainers and health professionals working in PHC facilities. Also accessed by managers.<br>• 40 000 of the first edition were printed: one for each facility trainer, seven for each rural facility and ten per urban facility.<br>• 40 000 additional copies were printed in 2021/22 and distributed to every health centre, ten per health centre.	Localised from PACK Global	Published 2017 (edition 1), updated 2019 (edition 2) and 2021 (edition 3).
**EPHCG guide – App version – Android and desktop applications**	Developed to circumvent printing costs and make the EPHCG guide more widely available.	The same clinical content as the hard copy version (see above) arranged in interactive mobile and desktop friendly format.	• The App version was developed at the request of health professionals using the EPHCG hard copy version and is the preferred format for those with access to internet and desktop or android devices.<br>• 10 000+ downloads recorded on Google Play store since launch.	Local innovation	The App version was launched January 2021 (Edition 2) and updated in June 2022 (Edition 3).<br><br>The Desktop application launched in 2023 (Edition 3).

**EPHCG-linked patient information booklets**[i]	To strengthen self-management for people with NCDs and mental health conditions treated through the EPHCG.	• Patient information booklets for NCDs and mental health conditions were adapted from those developed for PACK in South Africa.<br>• Based on EPHCG guide patient education recommendations but with more detail and design features suitable for non-health professionals.<br>• The focus was on clear information about the condition, treatment and self-management activities that could help outcomes.<br>• Translated into local language and illustrations contextualised to ensure user understanding.	• Designed to be used by people with NCDs and mental health conditions as self-management guides.<br>• Also designed for health professionals to orientate people (especially those unable to read) on self-management practices for chronic conditions.<br>• Budget restrictions have limited print runs and the booklets were distributed as soft copies to regional health bureaus.	Localised and de novo development	Developed in 2021.
**EPHCG training materials and programme**					
**Training curriculum**	To familiarise users with the structure and features of the EPHCG guide while focusing on clinical priorities and potential systems bottlenecks in order to embed its use during clinical practice.	The curriculum is based on clinical case scenarios which were adapted from the PACK training modules. It includes 12 clinical case scenarios that progress from simple to complex and cover clinical conditions commonly encountered in primary care, including communicable diseases, respiratory conditions, cardiovascular risk, diabetes and hypertension, tuberculosis, women's health, and mental health conditions.	• Clinicians working in primary health care facilities.<br>• Also accessed by trainers and managers.	Localised from PACK Global	2017/18
**Master Trainer Manual**	To support master trainers to deliver a facility trainers' training workshop and coordinate programme implementation at regional, zonal and woreda level.	• Programme for a four-day facility trainers' training workshop.<br>• Outline of Master trainer role to support EPHCG implementation.<br>• Designed to equip trainers and programme managers to supervise and monitor EPHCG programme implementation.	Master trainers	Localised from PACK Global	2017/2018

**Facility Trainer Manual**	To guide facility trainers to deliver onsite training sessions to health professionals	• Curriculum and programme for on-site facility EPHCG training sessions.	Facility trainers	Localised from PACK Global	2017/2018
**Training resources**	To support interactive sessions, allowing trainees to easily grasp EPHCG concepts in a fun, practical and visually compelling way.	The training resources include a board game, images of case scenarios depicted in a waiting room scene, and an infographic describing the EPHCG programme. The board game is designed to familiarize trainees with the features of the EPHCG guide before embarking on case scenarios.	• Trainers and trainees at each level of the training.<br>• The same number of these materials have been printed and distributed as the EPHCG guide.	Localised from PACK Global	2017/18
**Online training platform**	To enable provision of EPHCG training to remote health workers, reduce training costs, streamline EPHCG guide update trainings, standardize the training curriculum and to enable the training of new recruits when it is impractical to organize onsite sessions for fewer than 3 participants.	A web-based learning management system (LMS) hosted on the MOH eLearning platform that contains the EPHCG curriculum arranged in modules covering an introduction, the 'Assess, Advise, Treat' approach to a consultation and communication skills. The LMS has the capacity to register user details, monitor training progress, collate scores achieved for each module, provide discussion forums and award completion certificates and CPD points.	Health workers in facilities.<br>Since its launch, 482 trainees clustered in 21 groups have registered to take the EPHCG online facilitated training. In addition, 34 individual learners have registered and completed self-paced online training.	Local innovation	March 2023
**EPHCG online facilitators' manual**	To guide EPHCG online facilitators to facilitate online training sessions and to monitor training progress.	Contains the programme for a three-day workshop to capacitate EPHCG Master Trainers to provide EPHCG online training facilitation. This includes supporting online registration, monitoring training progress and opening discussion forums to encourage sharing of EPHCG training experiences.	MOH master trainers from all regions and city administrations. A total of 49 master trainers took the three-day Online training facilitators workshop.	Local innovation	March 2023?

**Primary health care clinical communication skills training manual and training programme**[ii]	To support health professionals to deliver person-centred care while using the EPHCG guide.	• Four facility-based training sessions designed to follow EPHCG training.<br>• Expanding on the brief clinical communication skills section in EPHCG training, sessions cover holistic assessment, healthcare worker wellness, managing emotions and promoting self-management.<br>• Accompanying videos illustrate competencies for person-centred care to support EPHCG delivery in the context of continuing care.[iii]	• Designed for health professionals in health centres.<br>• Training delivered with the quality improvement training.<br>• At national level, two rounds of Master Trainer workshops have been conducted with 47 regional and zonal health bureau health center service team experts. Cascade delivery of training will commence once budget is secured.<br>• 23 facility trainers and 88 health professionals in 16 health centres have received the training as part of the ASSET study.	Adapted from a training package developed in South Africa to support nurses to deliver person-centred care for patients with chronic diseases.[iv]	September 2021
**EPHCG-linked quality improvement training programme**[v]	To equip health professionals in health centres with skills and tools to perform quality improvement initiatives while implementing EPHCG, focusing on NCDs and mental health conditions.	• Seven weekly sessions developed using a the EPHCG cascade model of training and implemented in the facility with the full clinical team following completion of EPHCG training.<br>• Quality improvement projects are designed and implemented alongside the quality improvement on-site trainings, allowing for learning in practice.	• Health professionals in health centres.<br>• Training delivered with the clinical communications skills training.<br>• At national level, two rounds of Master Trainer workshops have been conducted with 47 regional and zonal health bureau health center service team experts. Cascade delivery of training will commence once budget is secured.<br>• To date, 16 master trainers, 35 facility trainers and 66	Local innovation	May 2021

			health professionals in 16 health centres have received the training.		
**Implementation tools and strategies**					
**EPHCG Implementation Manual**[vi]	To standardize EPHCG programme implementation across all health centres. The manual also addresses a monitoring framework for programme managers at all levels.	• Provides the EPHCG implementation framework from national to health facility levels.<br>• Contains various tools to support and standardise implementation: checklists for internal and external supportive supervision, EPHCG readiness checklists, implementation standards, mentorship guides, clinical audits, and a monitoring & evaluation framework.	Health centre staff and management, mentors, and supervisors at each level.	Local innovation	Developed in 2019 and updated in August 2021.
**EPHCG implementation support strategies**	To support the EPHCG training cascade and to sustain *woreda* and facility level motivation to continue with EPHCG programme implementation	• Supportive supervision and mentorship from *woreda* health offices and catchment hospitals. In Addis Ababa, sub-city health office staff were tasked with supervising and supporting health centres not under their jurisdiction to ensure fairness and sharing of experiences among health centres.<br>• A mechanism using EPHCG programme indicators to select best performing health centres from each region and recognise their efforts.<br>• Experience sharing visits among health centres from various regions at the best performing health centre (see box 1).<br>• A messaging platform (Telegram) was utilised to follow regional training rollout, share health	Health centre staff receiving EPHCG training and implementing the programme	Local innovation	2017/18

		centre experiences, provide weekly updates of resources and guidance, and provide a forum for questions.<br>• Health centre best-practice documentary videos disseminated through Telegram to regional health bureaus demonstrate the achievements of best performing health centres including their implementation of the EPHCG programme.[vii]<br>• Community-level awareness campaigns to generate community level support for EPHCG implementation utilised daily health centre health education sessions, print media, and pre-existing town hall meetings.			
**Monitoring and evaluation**					
**EPHCG Training record**	Monitors adherence of health centres to training model at facility level	• Paper-based recording system that logs each onsite training session, attendance register, and the curriculum covered in that session.<br>• Online training monitoring is situated within the learning management system (LMS). The LMS awards certification based on trainees' completion of the curriculum and performance on quizzes.	• Records are completed by facility trainers and trainees, authenticated by the facility administrator and filed in the health facility record after session completion. Supervisors and mentors check this record to ensure the correct steps were followed.<br>• Online session facilitators can monitor online trainee activity; users with higher level access can review analysed data, including number of completed training sessions and number of trainees by region or health facility.	Localised from PACK Global	2017/18

**EPHCG informing Clinical Audit Quality Indicators**	• Provides a standard of care for clinical auditing, against which health centre clinical practices are measured.<br>• To standardize EPHCG implementation across all health centres and to improve health worker adherence to the EPHCG guide.	• The MoH Ethiopian Health Centre Clinical Audit Guide and Tools,[viii] measures adherence of clinical practice against the standards of care provided by the EPHCG guide for adults and children over 5 years.<br>• The National Health Insurance Agency adopted the EPHCG guide as an audit tool to determine health centres' eligibility for reimbursement for services provided.	The Ethiopian Health Centre Clinical Audit Guide and Tools audits various health centre departments of care against standards of care set by the EPHCG guide and the Integrated Management of Newborn and Childhood Illness (IMNCI) guideline. It has been tested in 50 health centres with a plan to scale up to all health centres. For example, 19 patient charts are randomly selected to check whether diagnoses, appropriate investigations, routine care and counselling were done based on EPHCG guide recommendations. Disease-specific audits for tuberculosis and NCDs are also performed using EPHCG as a standard of care.	Local innovation	2022

[i] Center for Innovative Drug Development and Therapeutic Trials for Africa (CDT-Africa). Patient Information Booklets for Non-Communicable Diseases and Mental Health Conditions https://www.cdt-africa.org/index.php/projets/completed/asset
[ii] https://www.cdt-africa.org/index.php/projets/completed/asset
[iii] https://www.youtube.com/playlist?list=PLZKVJcLxnITLh_DeoYqI03ziCuizaLbE1
[iv] Change management for nurse-led chronic care manual. 2015 edition, Centre for Rural Health, University of KwaZulu-Natal. www.crh.ukzn.ac.za
[v] Federal Democratic Republic of Ethiopia Ministry of Health. Quality improvement Training for Primary Health Care Units https://www.cdt-africa.org/index.php/projets/completed/asset
[vi] International Institute for Primary Health Care - Ethiopia (IPHCE). EPHCG Implementation Manual 2019. http://196.189.110.22/handle/123456789/629
[vii] Health Centre Best Practice documentary videos https://www.youtube.com/watch?v=TSafJIAnKP4; https://www.youtube.com/watch?v=7aMhuzxGGpg

[viii] Ethiopian health center clinical audit guide and tools; November 2021. https://pdf.usaid.gov/pdf_docs/PA00ZS5H.pdf
